# Arabidopsis Plasmodesmal Proteome

**DOI:** 10.1371/journal.pone.0018880

**Published:** 2011-04-20

**Authors:** Lourdes Fernandez-Calvino, Christine Faulkner, John Walshaw, Gerhard Saalbach, Emmanuelle Bayer, Yoselin Benitez-Alfonso, Andrew Maule

**Affiliations:** 1 John Innes Centre, Norwich Research Park, Colney, Norwich, United Kingdom; 2 CNRS - Laboratoire de Biogenèse Membranaire, UMR5200, Bordeaux, France; Deutsches Krebsforschungszentrum, Germany

## Abstract

The multicellular nature of plants requires that cells should communicate in order to coordinate essential functions. This is achieved in part by molecular flux through pores in the cell wall, called plasmodesmata. We describe the proteomic analysis of plasmodesmata purified from the walls of *Arabidopsis* suspension cells. Isolated plasmodesmata were seen as membrane-rich structures largely devoid of immunoreactive markers for the plasma membrane, endoplasmic reticulum and cytoplasmic components. Using nano-liquid chromatography and an Orbitrap ion-trap tandem mass spectrometer, 1341 proteins were identified. We refer to this list as the plasmodesmata- or PD-proteome. Relative to other cell wall proteomes, the PD-proteome is depleted in wall proteins and enriched for membrane proteins, but still has a significant number (35%) of putative cytoplasmic contaminants, probably reflecting the sensitivity of the proteomic detection system. To validate the PD-proteome we searched for known plasmodesmal proteins and used molecular and cell biological techniques to identify novel putative plasmodesmal proteins from a small subset of candidates. The PD-proteome contained known plasmodesmal proteins and some inferred plasmodesmal proteins, based upon sequence or functional homology with examples identified in different plant systems. Many of these had a membrane association reflecting the membranous nature of isolated structures. Exploiting this connection we analysed a sample of the abundant receptor-like class of membrane proteins and a small random selection of other membrane proteins for their ability to target plasmodesmata as fluorescently-tagged fusion proteins. From 15 candidates we identified three receptor-like kinases, a tetraspanin and a protein of unknown function as novel potential plasmodesmal proteins. Together with published work, these data suggest that the membranous elements in plasmodesmata may be rich in receptor-like functions, and they validate the content of the PD-proteome as a valuable resource for the further uncovering of the structure and function of plasmodesmata as key components in cell-to-cell communication in plants.

## Introduction

An important goal in plant biology is the identification of the proteome of subcellular components and compartments. These data provide the foundation for functional studies involving additional and complementary approaches, e.g. cell biology and genetics. An excellent example of such a goal is the proteome of plasmodesmata (PD). PD are membrane-rich pores that bridge the relatively rigid cell wall to connect adjacent plants cells. They provide routes for the diffusion of small molecules from cell to cell, and for the specific trafficking of larger proteins and nucleic acids that collectively contribute to the regulation of development, growth and defence [1], [2]. Despite their importance in these fundamental processes, PD have remained recalcitrant to structural and functional dissection. Indeed, although they were first observed by Tangl in plant tissues in 1897, to date we know of only a handful of proteins that show a stable physical association with PD (reviewed in [3], [4]). Based conceptually upon the number of proteins associated with the nuclear pore complex, which has comparable functions in the translocation of small and large molecules between cellular compartments, we and others [3], [4] have speculated that PD might contain many tens of proteins involved in their architecture and operation.

**Figure 1:**
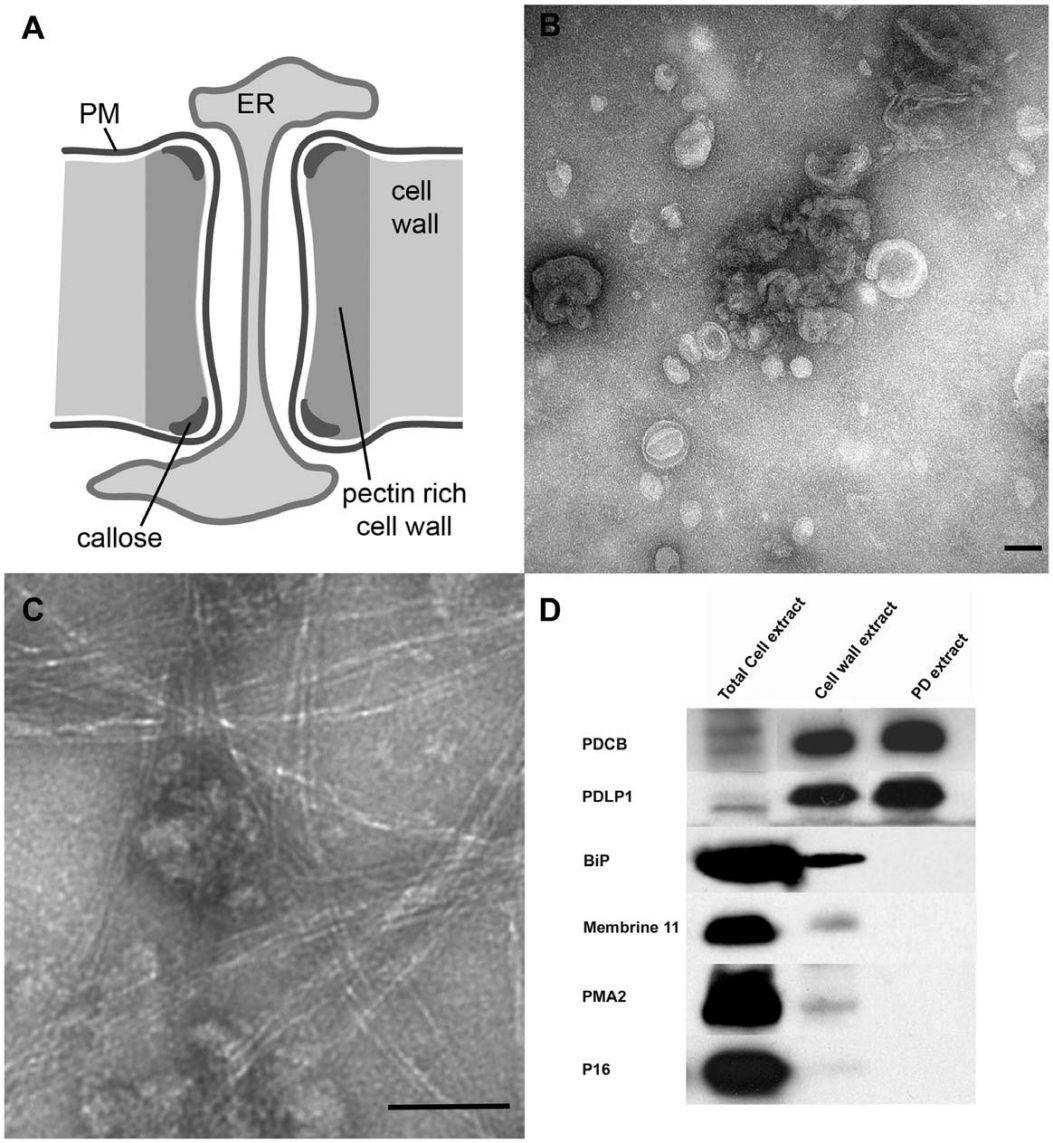
Isolation of plasmodesmata. The basic structure of plasmodesma (PD) is illustrated in Panel A. In addition to the key physical elements of PM, ER, desmotubule in the wall, a speculative arrangement of actin spiralled around the desmotubule is shown. Panel B shows a negatively stained electron micrograph of membranous PD (pellet P2 in M&M) collected after release from the cell wall following cellulase digestion, while Panel C shows contamination of the PD with residual cell wall fibres, observed very occasionally. Scale bars  = 100 nm. Panel D – Immunoblot analysis of fractions harvested during PD isolation procedure. Proteins extracted from whole cells, cell walls (pellet P1 in M&M) and purified PD (pellet P2 in M&M) were analysed using antibodies to the PD marker PDLP1, BiP (ER), Membrine11 (Golgi), PMA2 (PM) and P16 (chloroplast thylakoid envelope). While PDLP was enriched through the isolation procedure, the other proteins diminished and were virtually undetectable in the final PD preparation. *Total cell extract*: proteins extracted from 6 µl of Arabidopsis cell suspension lysate (corresponding to 0.6 µl of purified cell wall). *Cell wall extract*: proteins extracted from 75 µl of purified cell walls (pellet P1). *PD extract*: proteins extracted from 375 µl of purified PD (pellet P2).

PD are formed during cytokinesis following trapping of parts of the endoplasmic reticulum (ER) in the developing phragmoplast (defined as primary PD); secondary PD are also formed post-cytokinetically across existing cell walls. The pore is lined by plasma membrane (PM) that is continuous between adjacent cells. The ER becomes tightly appressed into a central axial element (desmotubule) in which the ER lumen is much diminished. The surrounding cell wall is distinct in that it is pectin-rich and contains variable deposits of β1,3 glucan (callose) around the neck region of the PD channel (Fig. 1A). The current consensus is that the callose collar forms a sphincter that physically limits molecular flux through the inner pore [5]–[11].

The main hurdle to identifying PD proteins is their physical location embedded in the complex matrix of the cell wall being therefore refractory to simple biochemical isolation. This has spawned a number of alternative approaches with mixed successes [12]–[28]. Of these, the most successful have been the immunological detection of candidate proteins and proteomic approaches. The former has identified components of the cytoskeleton (actin, myosinVIII, centrin [14], [19], [22], [24], [26], [27], [29]), ER-located calreticulin [12], and remorin [23]. For the latter, cell walls have proven to be an effective fraction enriched for PD. Hence, a cell wall fraction from tobacco led to the identification of a PD-located kinase [20], and 1-D gel electrophoresis of salt-eluted proteins from maize mesocotyl cell walls, identified a class 1 41 kDa reversibly glycosylated polypeptide (^C1^RGP2), which associated with PD following ectopic expression as a fluorescently-tagged fusion protein [25], [28]. ^C1^RGP2 is also a Golgi-associated protein; no function has yet been identified [15], [28]. 2-D gel separations of sub-cellular fractions from two cell types of *Chara*, differentiated by the presence and absence of PD, also identified a tropomyosin-like protein and RGP2 as PD-located proteins [18], [30]. In a refinement of the earlier cell fractionation approaches, the Epel group [21] released PD from *Arabidopsis* cell wall fractions using cellulase, separated extracted proteins by 1-D gel electrophoresis, and identified a 45 kDa β-1,3 glucanase (named *A. thaliana* beta-1,3-glucanase_putative Pd-associated protein; AtBG_ppap) using in-gel proteolysis and ion-trap mass spectrometry.

In our previous work [13], we established the proteome for cell walls isolated from a culture of rapidly dividing *Arabidopsis* suspension cells, using two-dimensional liquid chromatography tandem mass spectroscopy (2D-LC MS/MS) of total extracted proteins. The proteome, which included secreted and non-secreted proteins, identified both known (e.g. AtBG_papp, RGP2, calreticulin) and unknown PD proteins. From a total of 89 PM-targeted membrane proteins lacking ER-retention signals [13], new PD proteins were identified and comprised two families of membrane-associated proteins, called PD-located proteins (PDLP; [31]) and PD-callose-binding proteins (PDCB; [11]). These were targeted to PD as fluorescently tagged protein fusions and modified molecular flux through the channel following altered protein accumulation. In this paper we describe the outcome of a combination of our previous strategy with that described by Levy et al [21], to purify and characterise PD from *Arabidopsis* suspension cells. After using a more sensitive nano-LC ion-trap MS/MS method, we report a list of identified proteins that best describes to date the structural and functional proteome of PD from *Arabidopsis*. The size and content of the list indicates that the very sensitive technologies still reveal the presence of contaminant proteins but also that the list contains many proteins with known or inferred association with PD. Also, again focussing on membrane-associated proteins and using subcellular targeting as the criterion, we report the identification of several new putative PD proteins. These include several receptor-like kinases and a tetraspanin and, together with the identification of receptor-like properties of the PDLP proteins [31], suggest that PD may represent a membrane domain rich in receptor functions.

## Results

### PD isolation

The value of proteomics is strongly correlated with the purity of the target in the samples analysed. For PD, this is a major challenge since the membrane-rich structures and callose collars are integral to the structure of the insoluble wall matrix. Previously, we used isolated cell walls from *Arabidopsis* suspension cultures as samples enriched for PD with respect to extraneous cellular components. To achieve a higher level of PD enrichment in this work, suspensions of cell walls were digested with a commercial unpurified cellulase preparation and the released membranous components collected by differential centrifugation. Cell wall digestion gave approximately 70% digestion of cell wall mass. This could not be increased by higher concentrations of enzyme or longer digestion periods. Addition of pectin degrading enzymes (e.g. polygalacturonase) reduced slightly the amount of residual cell wall but gave no measurable improvement in protein recovery (data not shown). Centrifugation of the digested mixture at ∼6000xg nevertheless separated the remaining visible insoluble material from small particulate material retained in the supernatant, which could be collected using higher speed centrifugation. Transmission electron microscopy of negatively stained samples of the smaller material revealed vesicle-like structures of 50–100 nm (Fig. 1B), which appeared to be composed of limited numbers of concentric membrane layers. Occasionally, samples were contaminated with residual fibrillar material, probably remnants of cell wall microfibrils (Fig. 1C). Immunoblot analysis of samples collected sequentially during the PD isolation procedure showed that the membranous sample was substantially free of contaminant proteins representative of the endoplasmic reticulum (BiP), plasma membrane (PMA2), Golgi (membrine11), and chloroplast (thylakoid P16), whilst showing a corresponding increase in the abundance of PDLP1 (Fig. 1D).

### PD Proteomics

As previously [13], we applied a LC-MS strategy to preparations of total protein extracted from purified PD; nano-LC-MS/MS experiments were performed on an LTQ-Orbitrap^TM^ mass spectrometer. Since the aim was to determine the total protein compliment, consecutive runs were made until the novel protein detection was minimized (i.e. close to - >95% saturation). This series of runs (13 in total) also included minor modifications to the sample preparation (e.g. protease digestion conditions and length of LC separation) and several biological and technical replicates. For any one condition, reproducibility between technical replicates was approximately 60–70% and between biological replicates, approximately 50–70%. Protein identification was achieved by reference to the TAIR 8 database using MASCOT, SEQUEST and SCAFFOLD software. Using the criteria of greater than 99.0% probability of correct protein identification, for proteins identified with at least two unique peptides, the total number of proteins identified from these samples was 1341 (Table S1). We refer to this as the PD-proteome.

### Analysis of the PD-proteome

**Figure 2:**
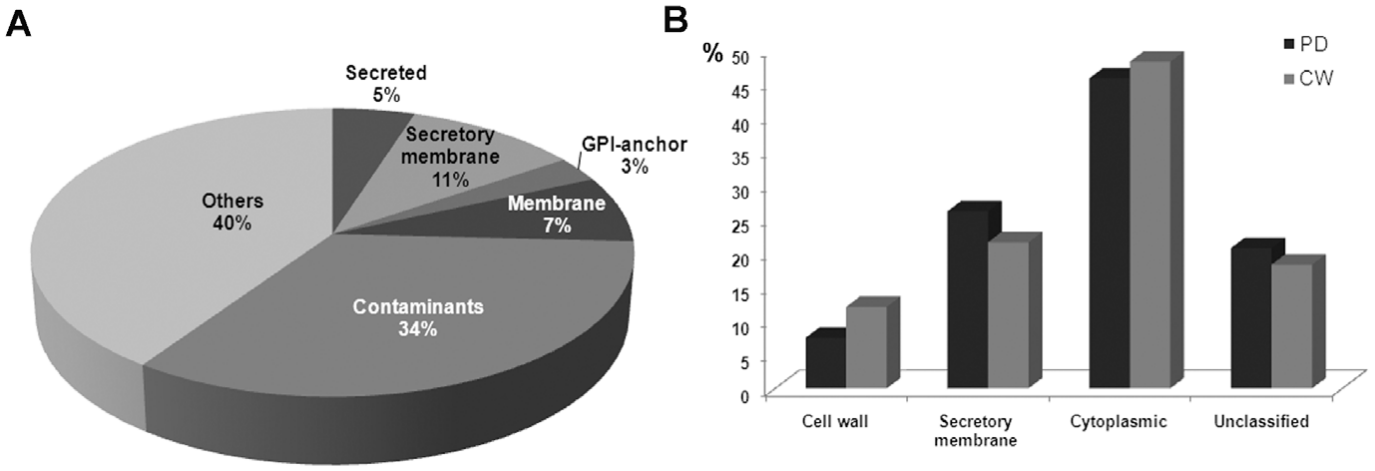
Analysis of the PD-proteome with respect to predicted subcellular localization and its comparison with the CW proteome. (A) The 1341 proteins of the PD-proteome were classified as secreted proteins, integral membrane proteins processed through the secretory pathway and targeted to Golgi, ER, PM and PD (‘secretory membrane’ proteins), GPI-anchor proteins, non-secreted membrane proteins, contaminant proteins and ‘others’, where others are proteins without membrane association and not predicted to be secreted. The contaminant category includes those proteins predicted to be targeted to chloroplasts, mitochondria and vacuoles. Transmembrane helices (using TMHMM [44]), signal peptides (SIGNALP [83] and SIGNALP-HMM [84]), subcellular location (TARGETP [85]) chloroplast transit peptides (CHLOROP [86]), and GPI-anchoring signals (DGPI [87]) were predicted using software as indicated. (B) GO ‘cellular component’ analysis was used to compare the PD-proteome with the previously reported [23] proteomic data for cell walls from *Arabidopsis* cell cultures (CW). The main cellular component categories; cell wall, secretory membrane, cytoplasmic and unclassified, proteins, were obtained using GO Slim. (The ‘secretory membrane’ class in B is equivalent to the same class in panel A, although it is defined using different software.) ‘Cytoplasmic’ includes plastid, chloroplast, mitochondria, nuclear, ribosome and cytosol proteins. Unclassified category contains other cytoplasmic, other intracellular and unknown cellular categories. Dark gray bars represent PD-proteome and light gray cell wall proteome.

To analyse the list of 1341 proteins, we used a number of bioinformatic tools, databases and literature sources, to obtain information about predicted subcellular localizations, and functional domains. Because of the high sensitivity of the Orbitrap mass spectrometer used, we anticipated the detection of PD proteins and a number of contaminant proteins. Given the acidic composition of the extracellular matrix and that our enriched PD fraction still contained very small amounts of undigested wall it was possible that cytoplasmic proteins, bound to the cell wall through ionic interactions, may also contribute to a pool of potential contaminant proteins. Although classifying a protein as a contaminant necessarily makes assumptions about the requirements for PD function, we judged that proteins from plastids, mitochondria, nuclei and some classes of cytoplasmic proteins would qualify. On this basis almost 35% of proteins were predicted to be contaminants, with chloroplast proteins being the most abundant (Fig. 2A). More than 10% (136) of all the proteins were ribosomal, which could have originated from cytoskeleton-bound polysomes anchored to the PM via actin filaments [32], [33].

From information recorded in the Plant Proteome Database (PPDB) we have observed that almost 75% of the proteins in the PD-proteome have been described previously in other proteomic studies. Approximately 40% are represented in PM proteomes [34]–[40] and 12% in cell wall proteomes [13], [41]–[43] (Table S2). In our and other proteomes, a significant number of proteins were recorded as being derived from multiple subcellular locations (Table S2).

The PD-proteome was analysed with respect to gene ontology (GO) terms for predicted functional categorization (represented by three main subcategories: ‘GO Cellular components’, ‘GO Molecular function’ and ‘GO Biological processes’) (Figure S1 and Table S3). To get a broad descriptive comparison with the Arabidopsis cell wall proteome, the Cellular Component subcategory of the GO was divided broadly into classes representing cell wall proteins, membrane proteins associated with the secretion pathway and potentially targeted to the cell periphery (Golgi, ER, PM and PD – secretory membrane proteins), cytoplasmic proteins (including, plastids, mitochondria, nuclei, cytosolic etc), and a group for which no prediction could be made (unclassified). These classes were compared with the cell wall proteome from *Arabidopsis* suspension culture cells [13] (Fig. 2B). GO classifications for single proteins may overlap between classes and so quantitative comparisons between classes could not be made. In comparison with our published *Arabidopsis* cell wall data, the PD-proteome showed a lower frequency of cell wall proteins and a higher proportion of membrane proteins, consistent with the removal of the cell wall by digestion before PD purification (Fig. 2B). Surprisingly, despite additional washes associated with isolating PD from digested cell walls the proportion of cytoplasmic proteins was similar to that found for purified cell walls. Overall, 33% of proteins present in the CW proteomic list were also present in the PD-proteome. The overlap contained 30% contaminants and ∼20% membrane-associated proteins. The latter group included known PD proteins, PDLP1 [31], and AtBG_papp (At5g42100; [21]).

**Figure 3:**
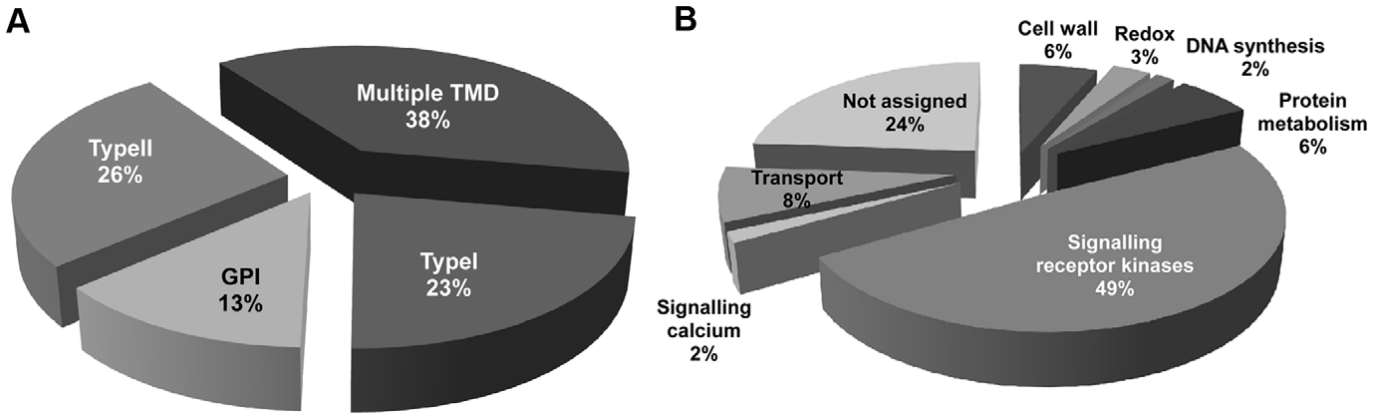
Analysis of membrane proteins targeted to the cell periphery. Membrane proteins from the PD-proteome were classified into four different categories (A): Type I membrane proteins (Type I), Type II membrane proteins (Type II), GPI- anchor proteins (GPI) and multiple transmembrane domain proteins (Multiple TMD). Panel B shows the predicted functional Mapman categories for the membrane proteins.

Since PD represent membrane-rich structures, we analysed the predicted membrane proteins in more detail with respect to their domain structures and functions. Based upon prediction softwares (TMHMM [44] and MEMSAT-SVM [45] and searches in publicly available algorithms (e.g. Aramemnon, TAIR, ExpaSy and NCBI sites), and excluding contaminant proteins, there are 279 membrane proteins (proteins with one or more transmembrane domains (TMD) excluding the hydrophobic signal peptide, or a GPI anchor) in the PD-proteome, 21% of total proteins. The group of membrane proteins potentially targeted to the PM (i.e. with a signal peptide but lacking ER-retention signal) was subdivided into type I, type II, multiple TMD, and GPI-anchored proteins (Figure 3A). The most abundant sub-grouping was the multiple TMD proteins (38%), followed by Type II proteins (26%), Type I (23%) and GPI-anchor proteins (13%). For the type I class of membrane proteins, 49% are receptor-like molecules (many being receptor-like kinases; RLKs) and only 11% are involved in transport (Fig. 3B).

As yet we can make few predictions as to the functional categories of proteins that might occur tightly associated with PD. Connected with our increased understanding of the nature of molecules that transit the channel we might anticipate the presence of chaperones for proteins and nucleic acids and the potential for activities to provide energy for the transport process. However, in reality we have very little idea as to which molecular functions should be present.

### Validation of the PD-proteome

**Figure 4:**
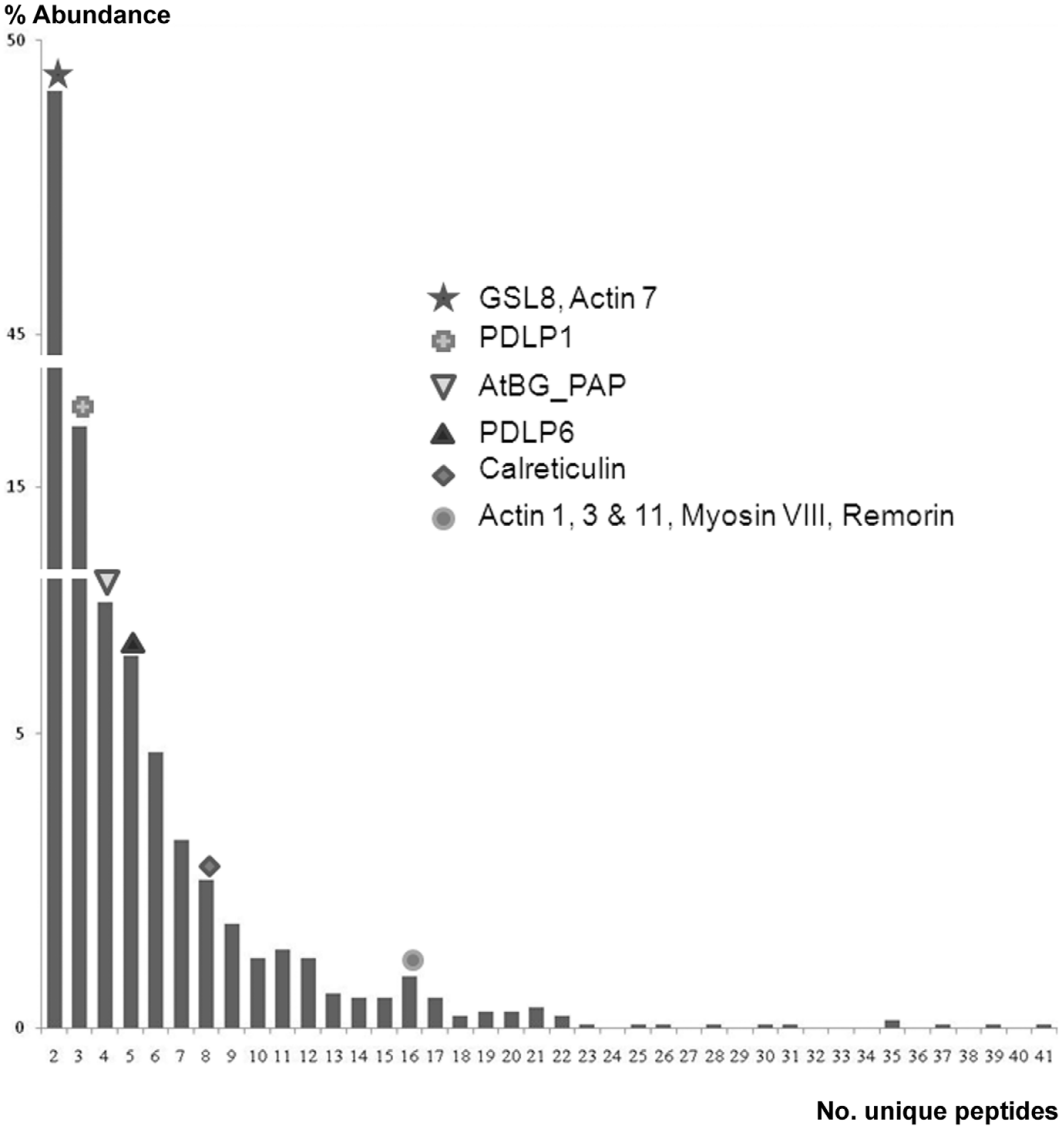
Distribution of known PD proteins in the total PD-proteome. In the hope of identifying potential PD proteins on the basis of the ease of proteomic detection (number of signature peptides), known PD proteins were placed upon a plot of the frequency of identified proteins against detected peptides. No positive correlation was found indicating that PD proteins are very variable in the abundance in PD.

The PD-proteome comprises 1341 proteins, a larger number than might have been predicted from parallels drawn between PD and the nuclear pore complex [46]. Despite the further purification of PD away from the cell wall, and the enrichment for membranous structures, we detected a significant number of apparent contaminant proteins derived from cytoplasmic components (including plastids, mitochondria, nuclei etc; Fig. 2A; Table S2). However, our proteome analysis was qualitative, not quantitative, and therefore does not reflect relative abundance. We reasoned that the low variability between replicates might be attributed to the large number of proteins detected with few (two or three) peptides, which in turn reflected the sensitivity of the Orbitrap detector and the presence of large numbers of proteins with low abundance. For these classes of proteins, detection might be stochastic and therefore variable between runs. Since the overall objective was to use purified PD to reveal the spectrum of novel proteins associated with PD, we also predicted that the proteome should contain known PD proteins and that these might be represented by the more abundant proteins. In theory, these should have been amongst those proteins detected with the largest number of tryptic peptides. The proteome contains a number of known PD proteins, i.e. PDLP1 and PDLP6 [31], β1-3 glucanase (AtBG_ppap; [21]) calreticulin [12], [47] and remorin [23]. Surprisingly, these showed no correlation with the number of detected peptides (Fig. 4) indicating that, despite PD purification, these proteins may have been very different in their abundance in our suspension cells relative to the tissues in which they were first identified.

**Figure 5:**
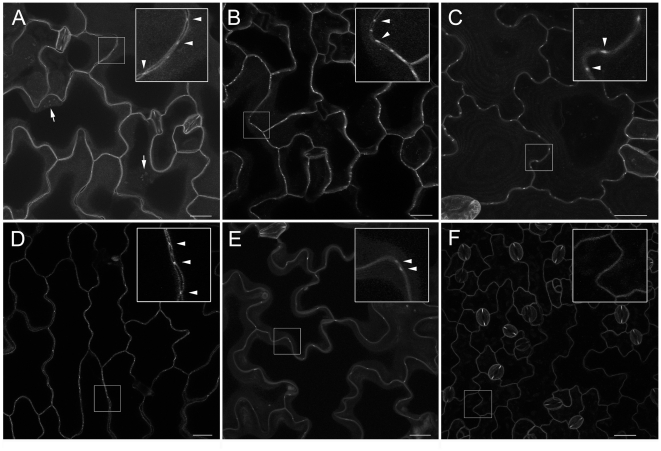
Novel PD proteins identified through their subcellular targeting. Transgenic expression of fluorescent fusion proteins and their targeting to puncta on the cell wall identified five new PD proteins. Panels A–F show projections of confocal laser scanning microscopy z-series of *Arabidopsis* leaf epidermal cells for YFP or mRFP fusions to the receptor-like kinases At1g56145(A, YFP), At4g21380(D, YFP), At5g24010 (E, RFP), and At5g59700 (F, YFP), hypothetical protein At3g15480 containing a DUF1218 domain (B, YFP) and a tetraspanin At3g45600 (C, YFP). PD localisation (arrowheads) is evident as punctae of fluorescence in the cell wall for images shown in A–E. For At1g56145 in (A), PD in pitfields at the epidermal-mesophyll boundary are visible (arrows). Panel F illustrates the targeting of a non-PD RLK At5g59700 to the PM. Bars  = 20 µm.

Previously, we focussed our attention on secreted membrane proteins lacking an ER-retention signal, mostly type I membrane- and GPI-anchored proteins, as an entry point for our search for novel PD proteins. For this work, in the absence of other indicators, we validated the authenticity of the PD-proteome by sampling a limited number of candidates from the class of membrane proteins theoretically targeted to the cell periphery for their potential to target to PD as fluorescent fusion proteins. We chose initially to sample a GPI-anchored β1-3 glucanase, following the precedent set by AtBG_ppap, and a number of the receptor-like proteins since these were abundant in the PD-proteome and PDLP had established a precedent for receptor functions at PD [31]. Genes for six RLKs and the β1-3 glucanase were cloned and expressed, using the CaMV 35S promoter, as translational fusions to YFP or RFP following stable transformation into *Arabidopsis*. Subcellular localization in leaf tissues was examined by confocal laser scanning light microscopy. Fluorescent proteins that target PD show a punctate distribution of fluorescence along the wall of plant cells that reflect the distribution of individual PD channels or, more commonly, the distribution of groups of PD in pit fields. Typically, these fluorescent puncta show co-localisation with aniline blue staining that identifies the callose deposits in the near-cell wall. Cell walls surrounding epidermal pavement cells and the interface between the lower epidermal wall and the subtending mesophyll cell were examined. At this latter location a face-on view of PD clusters in pit-fields is possible. Of the seven sampled proteins, three showed a pattern of fluorescence consistent with PD targeting (Table 1; Fig. 5). Of the six RLKs, three showed uniform labelling of the PM (Table 1; Fig. 5 with PM labelling with At5g59700 illustrated as an example); At4g27300 was targeted to the ER. In contrast, a punctate pattern of fluorescence, often combined with PM labelling, was identified for three of the RLKs: At1gG56145, At4g21380, and At5g24010 (Fig. 5). In each case, the punctuate fluorescence pattern showed co-localisation with callose, revealed by aniline blue staining (Fig. S2). In some cases, maximum projections of CSLM image stacks revealed the connections with the subtending mesophyll cells (example illustrated for At1g56145 in Fig. 5A). These genes encode LRR class VIII RLK, an S-domain RLK and a Catharanthus roseus RLK1-like protein, respectively (For a review of RLK class structure see [48]). The β1-3 glucanase was targeted to the ER.

**Table 1:** Experimental localization of selected candidates from the PD proteome.

AGI	MW	Description	Localisation
AT1G56145	112 kDa	LRR RLK	PM and PD
AT1G73650	34 kDa	Hypothetical protein - predicted oxidoreductase	PM
AT3G15480	19 kDa	Hypothetical protein containing a DUF1218	PM and PD
AT3G25290	43 kDa	Auxin responsive family protein	PM and ER
AT3G45600	32 kDa	membrane protein of unknown function - tetraspanin	PM and PD
AT3G45970	29 kDa	expansin protein (ATEXLA1)	Apoplast and vacuole
AT4G16120	73 kDa	ATSEB1 – GPI anchored	ER
AT4G21380	96 kDa	S-domain RLK	PM and PD
AT4G27300	92 kDa	S-domain RLK	ER
AT5G14030	21 kDa	Translocon-associated protein beta (TRAPB) protein	ER
AT5G24010AT5G58090	92 kDa52 kDa	CrRLK-likeβ-1,3-glucanase – GPI anchored	PM and PDER
AT5G59700	92 kDa	CrRLK-like	PM
AT5G60320	75 kDa	lectin RLK	PM
AT5G61790	60 kDa	Calnexin1	ER

a LRR: Leucine rich repeat, RLK: Receptor-like kinase, DUF: Domain of unknown function, CrRLK: Catharanthus roseus RLK; ER: Endoplasmic reticulum, PD: Plasmodesmata; PM: Plasma membrane.

A random selection of eight candidates with diverse-predicted or unknown functions was also tested (Table 1). Except for expansin (At3g45970), which targeted to the apoplast and vacuole, most (5/8) targeted to the PM or the ER. Two were targeted to fluorescent puncta on the cell wall (Fig. 5B and C), and also co-localised with callose (Fig. S2). These were proteins encoded by At3g15480 and At3g45600. At3g15480 has three TMDs included in a recognised domain (DUF 1218), but with no assigned function. At3g45600 encodes a tetraspanin TET3; TET3 has four TMD domains and has been implicated in the formation of specialised domains (tetraspanin webs) on the PM of animal cells [49].

## Discussion

PD present particular challenges when it comes to their molecular characterisation. Their location, embedded in the cell wall matrix, their functional and structural diversity associated with different symplastic boundaries in complex tissues, and their essential nature in maintaining co-ordinated growth and development, makes their study recalcitrant to a range of biochemical and genetic approaches. We have found it effective to exploit the physical and developmental simplicity of rapidly dividing and readily dispersing suspension cells as a way of characterising relatively uniform populations of primary PD in purified cell walls [50]. PD released from cell walls after digestion of the wall matrix showed the membrane-rich nature of the structures, represented by the PM and ER components. Surprisingly, while immunoblot analysis revealed enrichment of PDLP1 in the purified PD fraction it did not detect significant amounts of marker proteins for PM or ER. This indicates that while the PM and ER provide membrane continuity between cells, the nature of these membranes within the PD might be distinct. The PM in PD has been defined elsewhere [23] as a domain with similarity to membrane rafts (or microdomains) characterised by the presence of remorin and GPI-anchor proteins that preferentially reside in sterol-rich membrane domains. The ER may also be distinct in that it is very tightly appressed, excluding the much of the lumen.

The low abundance of other marker proteins (for Golgi, chloroplasts etc. detected by immunoblotting) showed that the biochemical strategy followed was an effective method for purifying PD. It was surprising then that proteomics detected such a large number of proteins, many of which appeared to be contaminants (e.g. derived from other cytoplasmic compartments). One likely explanation is the higher sensitivity of the Orbitrap technical platform and the qualitative nature of the assay, where abundant and rare protein species are listed equally. An alternative factor, however, is related to our relatively poor understanding of the nature and operation of PD. Only a few proteins have been shown to reside in PD and very few are believed to be uniquely associated with PD. Hence, although we have shown that PDLP1 is strongly localised to PD in leaves [31], we also find that it has a more dispersed localisation pattern in roots (unpublished data). Also, membrane proteins such as PDLP may arrive at PD via the secretory pathway [51] and therefore associate with the ER and Golgi in transit. Calreticulin and ^C1^RGP2 are targeted to PD but are also associated with the Golgi [25]; remorin is similarly found in PD but is also distributed in patches along the PM [23]. In addition to the physical association with PD, there is a much larger selection of proteins that have a functional association with PD but are found predominantly at other subcellular locations. For example, the RNA helicase proteins ISE1 and ISE2, which both affect trafficking through PD, and PD ontogeny [52], are found in mitochondria [53] and cytoplasmic RNA granules respectively [54]. Also THIOREDOXIN-m (TRXm), which profoundly influences PD gating and development, is a plastidial enzyme [55]. It remains possible that in particular cell types such proteins may have a transient association with PD. Lastly, non-cell-autonomous proteins (e.g. some transcription factors; [56]) may have a transient interaction with PD and may be captured when cells are plasmolysed prior to cell wall and PD isolation. In summary, there is no *a priori* reason why any protein in the PD-proteome list should be counted initially as irrelevant in the context of PD structure/function, although some (e.g. ribosomal proteins) intuitively may be less likely candidates.

Encouragingly, the PD-proteome list contains a number of the proteins, or likely orthologous proteins, for which experimental evidence shows a physical protein association with PD; identification of true orthologues, however, must remain speculative pending appropriate experimental data (Table 2). Hence, PDLP1 and PDLP6, AtBG_papp, calreticulin, remorin, type III peroxidases, several actins, and myosinVIII were identified in purified PD. Additional proteins, identified using complementary biological/experimental systems, have functions in common with representatives in the PD-proteome. For many of these, evidence for physical association with PD may not have been established. Hence, callose synthases, pectin methyl esterases, eIF4A, acid phosphatases, HSP70 were also identified (Table 2). A more useful analysis, however, is achieved by focussing on proteins with common biological roles.

**Table 2:** List of previously described PD proteins and their related proteins in the PD-proteome.

Std. Annotation	Acc. No.	No. Unique peptides	% Protein Coverage
**Proteins and orthologous proteins of known PD proteins**			
PDLP1	At5g43980	8	28.4
PDLP6	At2g01660	4	12.8
AtBG_PAP	At5g42100	5	19.6
Callose synthase 10 (GSL8)	At2g36850	16	11.5
Actin 1	At2g37620	2	42.4
Actin 3	At3g53750	2	42.4
Actin 7	At5g09810	16	55.7
Actin 8	At1G49240	6	48.5
Actin 11	At3g12110	2	48.8
Myosin VIIIA	At1g50360	2	3.12
Myosin IXK	At5g20490	3	3.69
**PD-related proteins with functions in common with representatives in the PD proteome**			
Calreticulin	At1g56340	3	8.25
AtPME1 (Pectin methyl esterase)	At1g53840	2	4.44
Pectinesterase putative	At2g47030	2	3.06
AtPME26 (Pectin methyl esterase)	At3g14300	2	3.1
Pectinesterase putative	At4g19410	2	10.2
Pectinesterase putative	At5g45280	2	6.22
AtPAP10 (Purple acid phosphatase)	At2g16430	20	53.6
AtPAP14 (Purple acid phosphatase)	At2g46880	5	16.1
Acid phosphatase class B	At1g04040	14	52.4
Acid phosphatase class B	At5g44020	7	40.8
HSC70.1 (Heat shock cognate 70)	At5g02500	22	39.2
HSC70.3 (Heat shock cognate 70)	At3g09440	5	27.6
CalS1 (Callose synthase 1)	At1g05570	2	8.26
ATGSL5 (Glucan synthase-like)	At4g03550	11	7.81
Glycosyl hydrolase 17 protein	At3g55430	2	6.46
eIF4A-1	At3g13920	2	29.4
eIF4A-2	At1g54270	15	36.7
UDP-glucoronosyl (glycosil transferase)	At3g46650	2	8.22
UDP-glucoronosyl (glycosil transferase)	At4g14090	2	5.26
AtPer12 (Class III peroxidase)	At1g71695	5	18.4
AtPer30 (Class III peroxidase)	At3g21770	2	10.9
AtPer44 (Class III peroxidase)	At4g26010	6	21.3
AtPer45 (Class III peroxidase)	At4g30170	3	10.2
AtPer57 (Class III peroxidase)	At5g17820	7	32.3
AtPer69 (Class III peroxidase)	At5g64100	2	9.37
Thioredoxin H3	At5g42980	2	28
Thioredoxin H5	At1g45145	2	16.9
DUF26 domain proteins			
PDLP1	See above		
PDLP6	See above		
Protein kinase	At1g70520	2	4.93

Callose deposition and turnover in the near-cell wall is central to the regulation of PD size exclusion limit (SEL). Hence, some β1,3 glucanases have a physical association with PD. Callose synthase 10 (GSL8) is specifically involved in callose deposition at PD [7]. The PD-proteome contains callose synthases (At1g05570, At4g03550, At2g36850), β1,3 glucanases (AtBG_PPAP (At5g42100), At5g58090) and other enzymes described as participating in the callose synthase complex (UDP-glycosyl transferases, At3g46650 & At4g14090) [57].

We are also increasingly appreciating the importance of redox control in the regulation of callose at PD and its impact on cell-to-cell communication [53], [55]. While the size exclusion limit and development have been shown to be regulated indirectly by redox status mediated by proteins located in plastids (GAT1; [55]) and mitochondria (ISE1, [53]), a more direct effect mediated by PD-located type III peroxidases has been suggested [58]. Peroxidases have been found by immunolocalization in the vicinity of PD and their location correlates with the presence of H_2_O_2_ [58]. The PD-proteome includes several class III peroxidases (AtPer12, At1g71695; AtPer30, At3g21770; AtPer44, At4g26010; AtPer45, At4g30170; AtPer57, At5g17820; AtPer69, At5g64100) which potentially are candidates to function as ROS generators in PD.

A number of other proteins with the potential to regulate cell redox status are also found in the PD-proteome. The list is extensive and includes oxygenases, oxidases, oxidoreductases and thiol redoxins. For example, we found two type h thioredoxins (TRXH5; At1g45145 and TRXH3; At5g42980). Previous studies of some members of this family found that they interact with PD and a role in the cell-to-cell and systemic transmission of redox signals have been suggested [59], [60]. The finding of these proteins in the PD-proteome strengthens the hypothesis that cell redox homeostasis is important for PD formation and function.

Protein trafficking to and through PD requires the support of molecules with chaperone like activity. HSP70 homologues isolated from pumpkin [61] have been shown to contain a short variable region (SVR) at the C-terminus at which the lack of a threonin seems to be responsible for their translocation through the PD [61]. A closterovirus–encoded HSP70 homologue (HSP70h) is also essential for protein translocation through PD [62]; HSP70- Arabidopsis homologues AtHSC70.1 and AtHSC70.3 are present at the PD proteome (At5g02500 and At3g09440 respectively) and they also lack the threonine aminoacid at the SVR, showing higher homology with those pumpkin HSC70 proteins that are able to facilitate transport through PD. Very recently, a chaperonin protein was identified from a genetic screen for molecules that assist in the intercellular transport of homeodomain containing proteins (Dave Jackson, Personal Communication). This chaperonin is predicted to be the theta subunit of the heterometric TCP-1 complex involved in protein folding [63]. The same chaperonin (At3g03960) and other members of the TCP-1 complex (At1g24510 and At3g18190) are present in the PD-proteome suggesting that the complex might be, at least transiently, associated with the channel.

The identification of the receptor-like PDLP family of proteins as PD components [31] raises the interesting prospect that receptors with the potential to sense extracellular signals, through their extracellular DUF26 domains, may influence the extent and/or specificity of cell-to-cell communication through PD. Although PDLP proteins lack an integral symplastic signalling module (e.g. an active kinase domain) they could signal into the PD by interaction with partner molecules providing the ancillary function. DUF26 receptor-like kinases have been shown to be responsive to salicylic acid [64] and DUF26 kinases are present in the PD-proteome (PDLP1, PDLP6 and a novel receptor-like kinase, At1g70520), although At1g70520 has not yet been tested for PD-targeting.

Other RLKs are also present in the PD-proteome and a limited survey of the potential for some of these to be PD-located proteins has identified three (At1g56145, At4g21380 and At5g24010) that target to PD when expressed transgenically as protein fusions to fluorescent markers. These proteins represent three new PD proteins to add to the current very limited list of PD components. The frequency (from a very limited survey) with which these proteins were identified suggests that the PD may represent a receptor-rich domain and points to a previously unrecognised potential for cell-to-cell communication to be influenced by factors in the extracellular environment. Very recently, Jo *et al* [65] reported preliminary evidence for the existence of six RLKs at PD in rice suspension culture cells. These RLKs comprise two wall-associated kinases, a lectin kinase and three LRR-kinases. None of these kinases were direct homologues of the proteins identified in this study. However, they do reinforce the view that PD represent a receptor-rich domain. Unfortunately, there is no evidence in the literature or from public collections of experimental data to indicate what the ligands for any of these receptors might be.

From our sampling of the membrane complement of the PD-proteome we also identified At3g15480 and TET3 as novel PD proteins. At present there are no indications as to the function of the protein encoded by At3g15480. In contrast, tetraspanins have been proposed in animal systems to define PM microdomains, called tetraspanin webs [66]-[68]. If equivalent structures also occur in plants, this may further indicate that PM in PD has a highly specialised organisation. From work with remorin (also present in the PD-proteome), we know that the PM passing through PD may also contain membrane raft microdomains [23], [69] and it has been proposed that membrane microdomains may provide the correct environment for clustering of receptor-like activities [49], [66], [68], [70]. The ER membrane contained within the desmotubule may also be defined by the presence of specific proteins. Reticulons are proteins that are associated with ER morphology, specifically in the constriction of ER tubules [71] and the identification of reticulons B3 and B6 (At1g64090 and At3g61560) in the PD-proteome raises the hypothesis that these proteins play a role in the constriction of the desmotubule. Further fractionation of PD into its constituent membrane components (ER, PM and membrane rafts) would be a feasible practical strategy for more formal testing of these hypotheses.

### Value of the PD-proteome

PD have been notoriously difficult to dissect with respect to their protein constituents. The existence of actual, inferred and experimentally validated PD proteins in the PD-proteome is testament to its potential in helping to overcome this barrier to understanding the structure/function properties of PD. For our experimental validation we selected a subset of 15 proteins to test for subcellular targeting. This selection was not completely random so does not allow extrapolation to the wider range of proteins with respect to the abundance of actual PD proteins. Nevertheless, the frequency of new PD proteins is highly encouraging. Our experimental analysis focussed on membrane proteins although membrane proteins constituted only 21% of the total. Our definition of membrane proteins was one that required an integral association and it seems very likely that some non-membrane proteins or loosely associated membrane proteins could also reside in PD, especially if they form complexes with integral membrane proteins. The value of the proteome data is extended through the use of alternative sources of complementary data. For example, by using publically available resources for gene expression (http://atted.jp; [72]) and protein-protein interaction (AtPid, http://atpid.biosino.org/) data new functional networks of proteins can be proposed that raise testable hypotheses. In summary, this PD-proteome provides the community with a valuable resource for cross-referencing from other PD-related experimentation or for the generation of new hypotheses about the functioning of these important cellular structures.

## Materials and Methods

### Preparation of plasmodesmata

Cell wall fractions from a rapidly dividing *Arabidopsis thaliana* (ecotype Landsberg erecta) cell suspension cultures [50] were treated with cell wall-degrading enzymes as described by Levy et al. [21] with modifications. Briefly, purified cell walls [50] were digested (1 ml per g of cell culture) with 0.7% w/v of cellulase R10 (Karlan) in digestion buffer (10 mM MES, pH 5.5, 4.4% mannitol) [21] and a cocktail of protease inhibitors (Sigma) for 2 h at 37°C with 100 rpm shaking. After centrifugation at 5860 xg for 5 min at 4°C, the supernatant and pellet (P1) fractions were collected separately. P1 was washed in digestion buffer and the two supernatants combined before centrifugation at 75600 xg for 40 min. The pellet was washed (10 mM MOPS, pH 7.5, 4.4% mannitol) and the final pellet (P2) resuspended in a minimal volume of buffer.

### Immunoblot analysis

Proteins from total cell homogenates and PD fraction were directly solubilised by boiling in 1X Laemmli buffer [73] for 5 min. Proteins from suspension culture cell walls were extracted sequentially in aqueous- and phenol-based buffers, as described previously [50]. Precipitated proteins were recovered by centrifugation, washed twice with 100 mM ammonium acetate in methanol and four times with 80% acetone. The protein pellet was left to air dry, then resuspended into 1X Laemmli buffer for 5 min. Proteins were separated using 10% SDS-polyacrylamide gel electrophoresis then blotted to PVDF membranes and analysed with anti-serum specific for PDLP1 (1/1250; [31], immunoglobulin-binding protein (BiP) (1/8000; [74]), Membrine11 (1/4000; antibody provided by A.Hocquellet, L. Maneta-Peyret & P. Moreau.), plasma membrane H+-ATPase (PMA2) (1/16000; [75]) and P16 (1/20000; [76]). Specific binding was visualised by standard techniques.

### Proteomic analysis

The protein pellet following extraction from isolated PD was dissolved in either a minimal volume of 8M urea, 0.1 M Tris-HCl, pH 8, or 0.5% Rapigest (Waters), 50 mM ammonium bicarbonate. Rapigest samples were heated in a boiling water bath for 5 min. All samples were reduced, alkylated, and digested with trypsin according to standard procedures. Digestion was halted by addition of trifluoroacetic acid and 0.5%. Rapigest was removed according to the manufacturer's protocol. Samples, digested in urea, were purified using OMIX® C18 tips (Varian Inc., Santa Clara, USA) before loading to the nanoLC.

Nano-LC-MSMS experiments were performed on an LTQ-Orbitrap^TM^ mass spectrometer (Thermo Fisher Scientific Inc., Waltham, MA 02454, USA). For nanoLC, two different systems were used: an Accela^TM^ HPLC (Thermo) with a flow splitter or a nanoAcquity UPLC^TM^ (Waters, Manchester, UK). The LC systems were run at a flow rate of 250 nL min^−1^ and coupled to the mass spectrometer via an ion source (Proxeon, Odense, Denmark) with a nanospray emitter (SilicaTips^TM^, 10 µm, New Objective, Woburn, MA 01801, USA). Samples were dissolved in 0.1% TFA and, on the nanoAcquity system, peptides were trapped using a pre-column (Symmetry® C18, 5 µm, 180 µm ×20 mm, Waters) which was then switched in-line to an analytical column (BEH C18,1.7 µm, 75 µm ×250 mm, Waters). Other runs were performed with the Accela^TM^ HPLC (Thermo) equipped with a trap column (C18 *PepMap^TM^, Dionex*, Camberley, UK) and a self-packed analytical column (BEH C18, 1.7 µm, Waters, 75 µm ×200 mm). Peptides were separated and eluted with a gradient of 5–45% acetonitrile in water/0.1%formic acid at a rate of 0.2% min^−1^.

Mass spectrometry was operated in positive ion mode at a capillary temperature of 200°C. The source voltage and focusing voltages were tuned for the transmission of MRFA peptide (m/z 524) (Sigma-Aldrich, St. Louis, MO). Data-dependent analysis was carried out in Orbitrap-IT parallel mode using CID fragmentation on the seven most abundant ions in each cycle. Collision energy was 35, and an isolation width of two was used. The Orbitrap was run with a resolution of 30,000 over the range of m/z 350 to m/z 2000 with an MS target of 10^6^ and 1 s maximum scan time. The MS2 was triggered by a minimal signal of 2000 with an AGC target of 3×10^4^ ions and 100 ms scan time.

For selection of 2+ an 3+ charged precursors, charge state and monoisotopic precursor selection was used. Dynamic exclusion was set to 1 count and 30 s exclusion time with an exclusion mass window of ±20 ppm. MS scans were saved in profile mode while MSMS scans were saved in centroid mode.

Tandem mass spectra were extracted by BioWorks version 3.3.1 and mgf files were generated using a perl script (Matrixscience). All samples were analyzed using Mascot (Matrix Science, London, UK; version Mascot 2.2) and Sequest (ThermoFinnigan, San Jose, CA; version 27, rev. 13).

Both Sequest and Mascot were set up to search the TAIR8 (20080413, 33024 entries) database, and both searches were done with a parent ion mass tolerance of 5.0 ppm and a fragment ion mass tolerance of 0.50 Da. Iodoacetamide derivative of cysteine was specified in Mascot and Sequest as a fixed modification. Oxidation of methionine was specified in Mascot and Sequest as a variable modification. Trypsin was designated as the protease and up to two missed cleavages were allowed. Tair 8 uses database entries for the Col-0 ecotype. Although our biological source material was L-er ecotype, database entries for the L-er ecotype are substantially fewer. L-er was selected for the suitability of the suspension culture for the biochemical purification of cell walls. The small sequence differences between Col-0 and L-er could have resulted in slightly fewer peptides being identified but this was outweighed by the benefit of the utility of the L-er suspensions and the larger database resources for Col-0.

Scaffold (version Scaffold_2_04_00, Proteome Software Inc., Portland, OR) was used to validate MS/MS based peptide and protein identifications. Peptide identifications were accepted if they could be established at greater than 95.0% probability as specified by the Peptide Prophet algorithm [77]. Protein identifications were accepted if they could be established at greater than 99.0% probability and contained at least 2 identified peptides. Protein probabilities were assigned by the Protein Prophet algorithm [78]. Proteins that contained similar peptides and could not be differentiated based on MS/MS analysis alone were grouped to satisfy the principles of parsimony. To calculate false discovery rates (FDR), the file was loaded into Scaffold version 3.00.3, and with the specified settings a protein FDR of 0.1% and peptide FDR of 5.3 was obtained. This Scaffold file has been lodged with TRANCHE <1?show $60#?tbklnk=="web1"$62#>(https://proteomecommons.org/tranche/). RAW Mascot files have been lodged with TRANCHE, and data is available in the PRIDE database [79] (www.ebi.ac.uk/pride). The data was converted using PRIDE converter [80] (http://code.google.com/p/pride-converter).

### Protein sequence feature prediction

Feature predictions for protein sequences in the proteomic output were automated using local installations of several software packages and Perl scripts. Because of the importance to this study of identifying likely transmembrane domains we used two independent programs; TMHMM [44] for fast processing of candidates and then a second evaluation of TMHMM-positives using the MEMSAT-SVM [45] tool. This has been shown to be more accurate [45] but is more computationally intensive, relying on a PSI-BLAST [81] search (versus UniProt [82]). MEMSAT-SVM explicitly attempts to identify signal peptides, and in conjunction with results of SIGNALP(-HMM) [83], [84] these helped to highlight possible false-positive TM regions near the N-terminus. Both TMHMM and MEMSAT-SVM predict not only positions of TM-domains, but also their topology; the end of each predicted TM-segment is predicted to be ‘inside’ (cytoplasmic) or ‘outside’ (extracellular, or in the ER lumen depending on the context). Here, we use ‘Type I’ to denote those proteins with a single predicted TM domain with the N-terminus outside and ‘Type II’ to denote those predicted single-TM domain proteins with the N-terminus inside. ‘Multiple TMD’ denotes those with multiple TM domains. Additionally, we applied programs to predict subcellular location (TARGETP [85]) and chloroplast transit peptides in particular (CHLOROP [86]), and GPI-anchoring signals (DGPI [87]). We also used our own Perl script to search for C-terminal tetrapeptides (HDEL, KDEL, REEL) indicating possible ER-retention. For individual candidates, especially those with a GPI-anchor where fluorescent reporters were inserted internally in the coding region, additional information was collected using Aramemnon and tools available through TAIR, Expasy and NCBI.

We obtained further functional annotations of our dataset from the MapMan [88] and Gene Ontology (GO) [89] resources. Each protein was placed in one of the MapMan “bins”, using the online search facility of the Plant Proteome Database (PPDB, [90]). Note that terms attached by the GO Consortium to genes/proteins summarize what is known from published experimental and/or computational studies, as well as the results of automated electronic annotation. It is therefore possible for seemingly contradictory terms to be attached to the same protein, even when supported by experimental evidence (for example, when a protein has been identified in independent published studies of two different organelles). Assessing GO terms of our proteins is nevertheless useful for obtaining an overview of the functional and spatial profile of a large dataset. To this end, we used the GO Plant Slim developed by The *Arabidopsis* Information Resource [91], rather than the highly detailed, complete Gene Ontology when comparing proteome data sets.

### Comparison of the PD-proteome with other published proteomes

At a basic level, the online search facility of the Plant Proteome Database (PPDB, [90]) was used to compare proteins identified in the plasmodesmal proteome with proteins listed within the Proteomic Publications collection. For specific comparisons with our previously published [13] *Arabidopsis* cell wall proteome (89 secreted proteins from a total of 792 proteins) we compared amino acid sequences since the databases use different identifiers for the same sequence. We looked for matching proteins by aligning pairs of proteins between sets using NEEDLE from the EMBOSS package [92] with a conservative global pairwise identity threshold of 95%.

### Gene cloning and expression

Clones for the transient and transgenic expression of *Arabidopsis* genes were generated using Gateway technology (Invitrogen). Gene sequences were amplified by PCR using Phusion DNA polymerase (NEB) from a genomic DNA or cDNA made from the aerial tissues of *Arabidopsis thaliana* Col-0 plants, using Gateway adaptor primers; primer sequences are available upon request. Resulting DNA fragments were recombined into the entry vector pDONR207 (Invitrogen). The sequence of the resulting pDONR clone was verified by automated sequencing.

Validated entry clones were recombined with binary destination vectors pB7FWG2,0, pB7RWG2,0 or pB7YWG2,0 clone [93] providing expression from *Agrobacterium* T-DNA, using the cauliflower mosaic virus 35S promoter upstream of coding fusions to green fluorescent protein (GFP), red fluorescent protein (RFP) or yellow fluorescent protein (YFP), respectively. GPI-anchored proteins were tagged internally with m-Citrine following published protocols [94]. Binary clones in *Agrobacterium tumefaciens* GV3101 were used for plant transformation [95].

### Confocal microscopy

Plant tissue was imaged at room temperature using a Zeiss LSM510 confocal microscope with an Argon ion laser. GFP and YFP were excited at 488 nm, and the emitted light was captured at 495–520 nm and 525–650 nm respectively. RFP was excited using 561 nm and emitted light captured at 590–630 nm. Images were captured digitally and handled using the Zeiss LSM image browser software. For callose staining, seedlings or mature leaves were infiltrated with 0.1% aniline blue solution. Aniline blue fluorochrome was excited at 405 nm and emitted light captures at 420–480 nm. Sequential scanning was used to image aniline blue with YFP or RFP.

## Supporting Information

Figure S1 **Gene ontology (GO) terms for the predicted functional categorization of the PD-proteome.** The three main subcategories are represented: Cellular components (A), Molecular function (B) and Biological Processes (C).
(TIF)

Figure S2 **Colocalisation of fluorescent puncta with callose.** Leaf tissues stably expressing fluorescent protein fusions (left panel) were stained with aniline blue (centre panel) to identify sites of callose deposition. Colocalisation of the fluorescence (right panel) supports these fluorescent puncta as the location of PD on the wall. Similar patterns of staining were seen for proteins encoded by At1g56145 (A), At3g15480 (B), At3g45600 (C), At4g21380 (D) and At5g24010 (E). Bar =  10 μm.
(TIF)

Table S1 **Complete list of PD-proteome sequence identities (1341) with associated the proteomic information.** ^a^It should be noted that when paralogous proteins could not be distinguished, all were included.
(XLS)

Table S2 **PD-proteome with Mapman Bin functional categories and predicted information on subcellular localization and description of proteins in the Public Proteome collection (PPDB) (ProteomicsPub. Column).** a CHLOR =  Chloroplast protein; MIT =  Mitochondrial protein; VACUOL = Vacuolar protein; S = Secreted; SM = Secretory membrane; NSnoTM =  Nonsecreted no transmembrane protein; NSTM =  Non secreted transmembrane protein; GPI =  GPI anchor protein.
(XLS)

Table S3 **PD-proteome with Gene Ontology descriptions.** ^a^ Comp = Cellular component; Proc =  Biological processes; Func =  Functional categories. RAW Mascot files have been lodged with TRANCHE (https://proteomecommons.org/tranche/), and protein and peptide identifications with associated spectra have been lodged with ‘PRIDE’ (http://www.ebi.ac.uk/pride/easySubmitData.do).
(XLS)
