# Supplementary figures and images for: Arabidopsis Plasmodesmal Proteome
Source: PLoS One. 2011 Apr 20;6(4):e18880. doi: 10.1371/journal.pone.0018880 (PMC3080382; doi:10.1371/journal.pone.0018880)

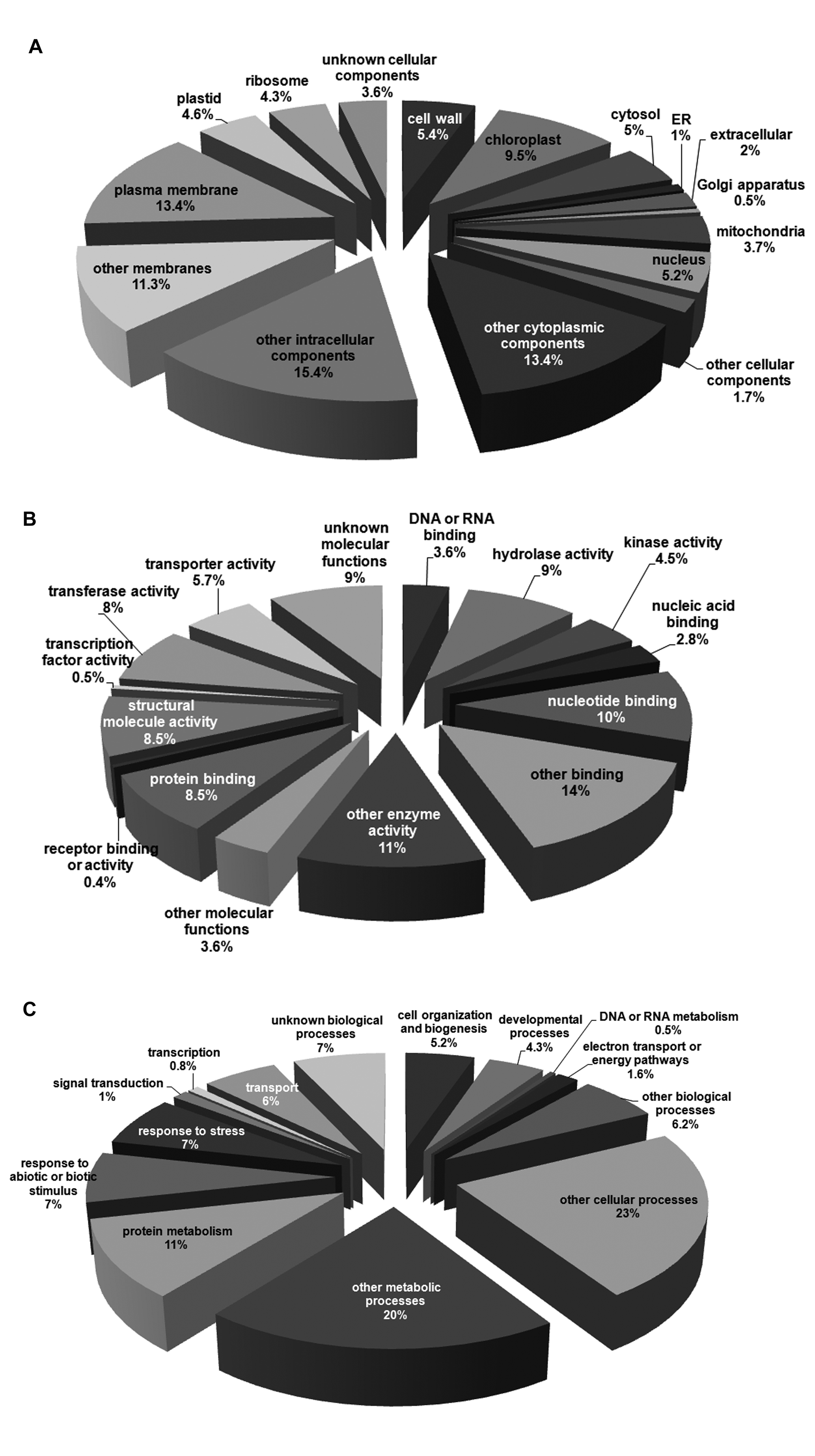

Supplement: Figure S1 — Gene ontology (GO) terms for the predicted functional categorization of the PD-proteome. The three main subcategories are represented: Cellular components (A), Molecular function (B) and Biological Processes (C). (TIF) [file pone.0018880.s001.tif]

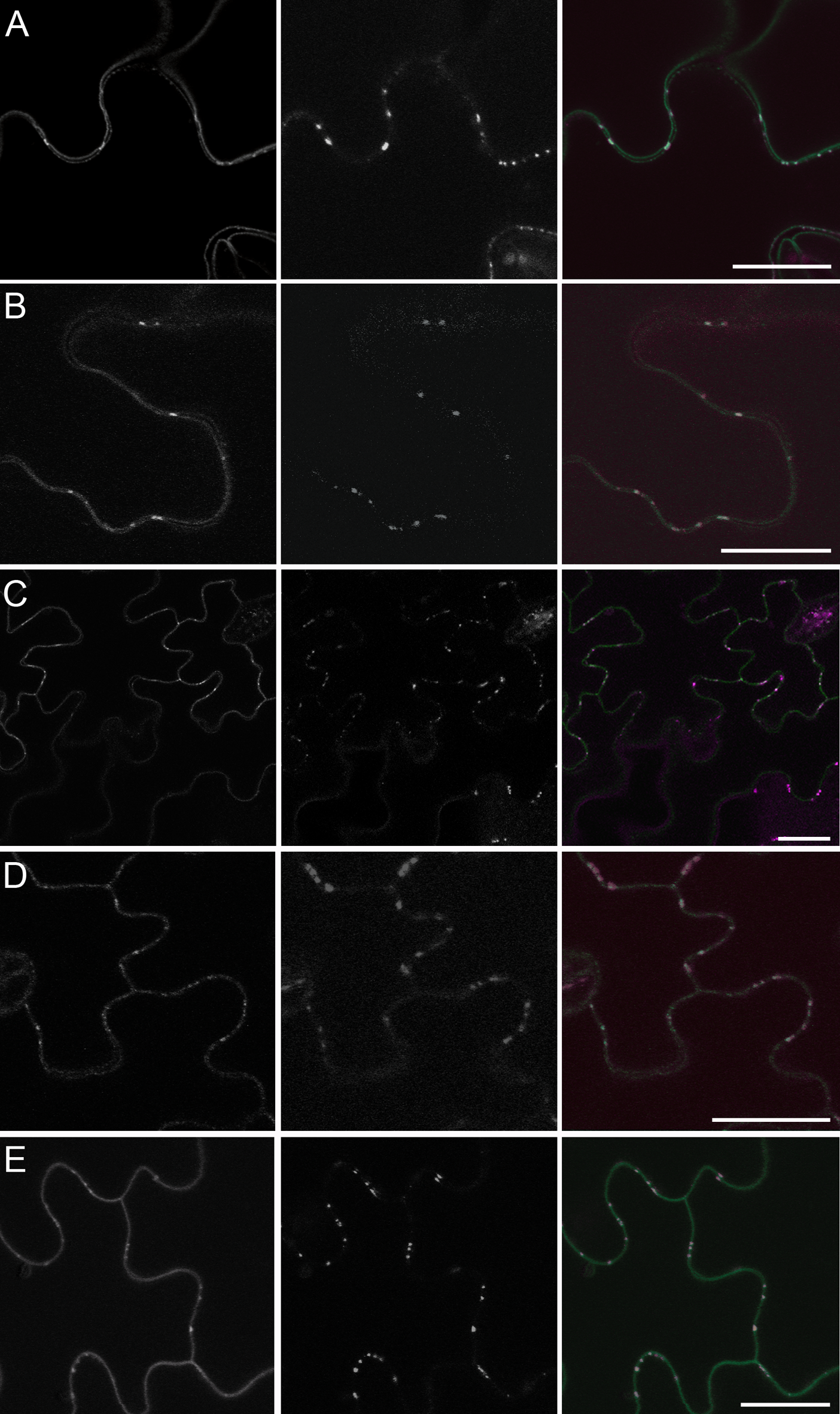

Supplement: Figure S2 — Colocalisation of fluorescent puncta with callose. Leaf tissues stably expressing fluorescent protein fusions (left panel) were stained with aniline blue (centre panel) to identify sites of callose deposition. Colocalisation of the fluorescence (right panel) supports these fluorescent puncta as the location of PD on the wall. Similar patterns of staining were seen for proteins encoded by At1g56145 (A), At3g15480 (B), At3g45600 (C), At4g21380 (D) and At5g24010 (E). Bar = 10 μm. (TIF) [file pone.0018880.s002.tif]
